# Reduction in the incidence of VAP and mortality rates in the ICU after implementation of hand hygiene education protocols and extensive ICU reconstruction work

**DOI:** 10.1186/cc9907

**Published:** 2011-03-11

**Authors:** K Filos, F Fligou, A Gotsi, D Velissaris, C Sklavou, M Marangos

**Affiliations:** 1 University of Patras, School of Medicine, Rion - Patras, Greece

## Introduction

Education in effective hand hygiene in the ICU is often neglected. The scope of this study is to detect the effects of an educational program on the incidence of VAP in a mixed ICU.

## Methods

Two groups of patients in two comparable time periods (9 months each) before and 6 months after implementation of various hygiene measures were analyzed. The measures implied: implementation of foot-operated hand washbasins, training in the effective use of hand washing followed by use of alcohol-based antiseptic dispensers near each ICU bed, and others. The diagnosis of VAP was by using clinical, microbiological, radiographic criteria, and by the CPIS index. Statistics was with ANOVA and x2.

## Results

Despite the comparable APACHE II scores at ICU admission (17.6 ± 6.5 vs. 18.1 ± 6.9) the two groups differed in variables as shown in Table 1. The incidence of VAP and mortality of Group 2 patients were significantly reduced. The RR of death in the control group was significantly increased (RR = 1.364, 95% CI: 1.055 to 1.763). The mortality of trauma patients in the protocol group was significantly lower (Group 1: 57.1% vs. 0% (Group 2), *P *< 0.05).

**Table 1:** Mortality in crude ICU patients and trauma patients with VAP

	Mortality of all patients (%)	Incidence of patients with VAP/mortality (%)	Incidence of trauma VAP patients/mortality (%)
Group 1 (*n *= 201, control)	44.3%^#^	10.4/67.6	15.9/57.1
Group 2 (*n *= 191, protocol)	32.5%**	5.2/40.0*	8.9/0*

## Conclusions

The implementation of protocols regarding hand hygiene by healthcare professionals in the ICU, together with a reconstruction, may lead to a significant reduction in the incidence of VAP and mortality both in the crude ICU patient population and in the subgroup of polytrauma patients.

